# Matrix metalloproteinases and their tissue inhibitors direct cell fate during cancer development

**DOI:** 10.1038/sj.bjc.6601327

**Published:** 2003-11-11

**Authors:** C V Hojilla, F F Mohammed, R Khokha

**Affiliations:** 1 Ontario Cancer Institute, University Health Network, Toronto, Canada

**Keywords:** MMP, TIMP, dissociation, apoptosis, proliferation, tumorigenesis

## Abstract

Matrix metalloproteinases (MMPs) were initially recognised for their extracellular matrix (ECM)-degrading capability during tissue remodelling. Their importance was further highlighted by their role in metastasis. Clinical trials have since evaluated the potential of MMP inhibitors as anticancer therapeutics, but without success. These initial studies point to the complex, multifunctional capacity of MMPs in cancer as shown by their function, not only as strident mediators of advanced malignancies, but also as effectors of early stage tumorigenesis. Research now shows that MMPs, and their tissue inhibitors, affect tumour initiation and growth through loss of cell adhesion, evasion of apoptosis, and deregulation of cell division. The extracellular nature of the metalloproteinase axis situates it as a master regulator of cell fate.

The extracellular microenvironment is a dynamic entity that dictates cell function and cell fate. While extensive research has established the importance of intrinsic cellular signalling pathways, the extracellular microenvironment is just as critical for governing cell fate and function. The extracellular microenvironment provides regulatory signals that affect important processes such as cell adhesion, differentiation, division, and apoptosis. Disruption of these functions can lead to acquisition of tumorigenic properties, such as loss of contact inhibition, aberrant cell division, and evasion of apoptosis.

Matrix metalloproteinases (MMPs) are essential regulators of the cell's microenvironment, through their control of extracellular proteolysis. Owing to their extracellular matrix (ECM)-degrading ability, the importance of MMPs during cancer progression was initially highlighted in the dissemination of cancer cells during metastasis. Since the ECM is a physical barrier, prevention of proteolytic breakdown by MMPs represented a key therapeutic target during tumorigenesis and many pharmaceutical companies became involved in developing MMP inhibitors as anticancer therapeutics. The failure of many clinical trials, thus far undertaken with broad-specificity inhibitors of MMPs, highlights the complexity of MMP biology. New research into the roles of MMPs reveals a much more fundamental involvement in normal cell function and in the initiation and promotion of tumorigenesis beyond merely cancer cell migration.

Matrix metalloproteinases represents a family of proteases that can process virtually any component of the ECM (Vu and Werb, 2000; Egeblad and Werb, 2002; Puente *et al*, 2003). They are Ca^2+^- and Zn^2+^-dependent proteases that are activated by the removal of an amino-terminal propeptide domain. Activation of MMPs is achieved either by autoproteolysis or processing by another MMP or a serine protease. While there are currently over 24 human MMPs and homologues from other species, MMPs were initially classified as collagenases, gelatinases, stromelysins, and matrilysins, based on their ECM substrate specificity. Current nomenclature employs a sequential numerical system as the list of substrates has grown and these are not restricted to ECM components. Based on shared functional domains, MMPs can be divided into eight groups, of which five are secreted and three are membrane-bound (Egeblad and Werb, 2002). Additionally, other classes of proteases that share the metalloproteinase domain with MMPs have been identified as ADAMs (A Disintegrin And Metalloproteinase) and ADAM-TSs (ADAM with thrombospondin domain) (Primakoff and Myles, 2000). Currently there are over 33 members of the ADAM family, which are cell surface-associated, and over 17 members in the ADAM-TS family, which are secreted proteins (Tang, 2001). However, not all of these have demonstrated metalloproteinase activity. While the ECM substrates of ADAMs are few and limited, they can proteolytically process a diverse group of cell surface proteins including ligands and their receptors.

The proteolytic activity of MMPs is regulated by a number of physiological inhibitors. The nonspecific MMP inhibitor *α*2-macroglobulin tethers MMPs to scavenger receptors, which are then irreversibly endocytosed. Specific inhibition is mediated by the Tissue Inhibitors of MetalloProteinases (TIMPs) (Vu and Werb, 2000; Egeblad and Werb, 2002). TIMP-1 through TIMP-4 are secreted inhibitors that selectively and reversibly bind MMPs in a 1 : 1 stoichiometry. Specific TIMPs also inhibit specific ADAMs. Small molecules that possess TIMP-like domains such as the carboxy-terminal fragment of the procollagen C-terminal proteinase enhancer protein and the NC1 domain of collagen type IV can also inhibit MMPs. REversion-inducing Cysteine-rich protein with Kazal motifs (RECK) have been identified as a membrane-bound MMP inhibitor (Oh *et al*, 2001). With the staggering number of MMPs and ADAMs, and the limited number of TIMPs and RECK, one can appreciate that a tightly regulated balance between proteases and their inhibitors must exist at any developmental and physiological stage. A disruption in this balance is observed in many pathological conditions such as arthritis, pulmonary emphysema, cardiovascular diseases, and cancer.

While it is established that MMPs degrade ECM proteins and therefore allow for cell migration, MMPs can also affect cell regulatory signals, directly or indirectly, within the extracellular microenvironment that impact intracellular signalling. In this review, we discuss how the proteolytic axis constituted by MMPs/TIMPs/ADAMs influences the basic cellular processes that underlie cancer development, namely cell dissociation, death, and division – the three D's of tumorigenesis.

## CELL DISSOCIATION

Cell adhesion is key to cellular organisation within a tissue. In addition to prescribing order to a group of cells, cell adhesion imposes a physical restriction that inhibits cell division when in contact with a neighbouring cell, a process known as contact inhibition. A key feature of transformation is the loss of cell contact inhibition and organisation. Cell adhesion is accomplished by cell–cell and cell–ECM contacts. Adherens junctions are among the many multiprotein adhesion complexes that mediate cell–cell contact. Upon cell contact, Ca^2+^-dependent homotypic engagement of cadherins occurs, which allows for the docking of other proteins such as catenins. This then acts as an actin cytoskeleton nucleation point (Christofori, 2003). Cell–ECM contact formation is mediated by the interaction of ECM components with integrin molecules. Upon binding, the cytoplasmic tail of the integrin acts as a docking point for other signalling molecules such as focal adhesion kinase (FAK) (Christofori, 2003). Engagement of integrins also allows for the assembly of actin cytoskeleton thus creating a link between the extracellular and intracellular environments.

Matrix metalloproteinases can influence cell adhesion by processing the extracellular components of cell–cell and cell–ECM contacts. Cleavage of integrins is demonstrated in nonmetastatic MCF-7 breast cancer cells. MMP-14-mediated processing of pro-alpha(v) integrin subunit results in the maturation of alpha(v)-beta(3) integrin facilitating invasion of these cells, without altering its interaction with ECM components. It has been proposed that the shift to invasive behaviour is due to increased activation in FAK signaling, which is implicated in numerous growth promoting pathways (Deryugina *et al*, 2002).

Cadherin-mediated cell–cell contacts are also subject to MMP processing. Upon TIMP-1-downregulation, fibroblasts become tumorigenic, at least in part, due to improper cell–cell and cell–ECM contact formation, thereby circumventing contact-inhibited growth (Ho *et al*, 2001). Upon treatment with MMP inhibitors, these fibroblasts revert to a normal phenotype by restoring adequate cell adhesion with increased localisation of *β*-catenin to cell–cell contacts and increased FAK activation (Ho *et al*, 2001). The loss of cell adhesion turns order into chaos. The shedding of adhesion molecules contribute to altered cellular organisation and architecture.

**Figure 1:**
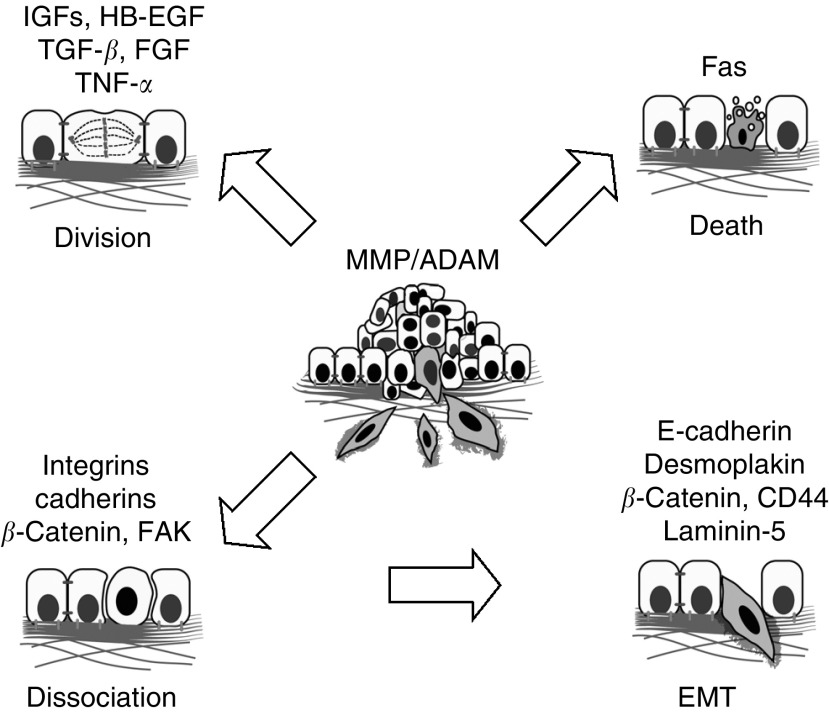
Perturbations in the extracellular proteolytic axis can lead to dysregulation of cell dissociation, cell death, and cell division, the three D's of tumorigenesis. Shown are the molecules currently known to be processed by metalloproteinases. These are linked to the loss of cell adhesion underlying loss of cell contact inhibition and EMT, evasion of cell death, and aberrant cell proliferation.

In addition to providing an initiating cue that can lead to perturbations in survival signals, cell dissociation can lead to changes in cellular differentiation pathways that further bestow malignant phenotypes to normal cells (see Figure 1

). A key differentiation event during cancer progression is epithelial-to-mesenchymal transition (EMT) (Birchmeier *et al*, 1996). During EMT, epithelial cells progressively acquire characteristics that transform them into a mesenchymal or fibroblast-like cell. This can also lead to increased extracellular proteolytic activity that facilitates migration (Birchmeier *et al*, 1996; Gilles and Thompson, 1996). The ability of MMPs to process cadherins contributes to the initiation of EMT. MMP-3 or MMP-7 triggers EMT by cleavage of E-cadherin (Noe *et al*, 2001). In these studies, the proteolytically processed fragment of E-cadherin may disrupt cell–cell contacts by interfering with the function of other full-length E-cadherin molecules. Furthermore, using mammary epithelial cells, induction of MMP-3 activity revealed a loss of desmoplakin and E-cadherin at cell–cell contacts, with a subsequent decrease of *β*- and *γ*-catenins suggesting a disruption in adherens junction formation (Lochter *et al*, 1997). In addition, an increase in the mesenchymal marker vimentin was concomitant with a downregulation of epithelial cytokeratins. Induction of MMP-3 also led to the upregulation of the mesenchymal growth factor, keratinocyte growth factor. Importantly, the molecular characteristics of EMT triggered by MMP-3 expression were not reversed by either removal of MMP-3 induction or addition of a broad-spectrum MMP inhibitor (Lochter *et al*, 1997). Activation and induction of other MMPs were also evident upon MMP-3 induction. These observations suggest that MMP-3 activity may lead to an irreversible cascade in the initiation of EMT.

The classically characterised role of MMPs in invasion and metastasis is a further step in EMT. In addition to increased MMP activity, MMPs become focused and localised during invasion. MMP-2, MMP-9, and MMP-14 congregate at the leading edge of a metastatic cell in order to facilitate confined and coordinated breakdown of the ECM barrier (Nakahara *et al*, 1997). Focused MMP activity may also promote migration in other ways. For example, cleavage of CD44 by MMP-14 results in increased motility in different cancer cell lines and mutation of the putative MMP-14 cleavage site in CD44 inhibits migration (Kajita *et al*, 2001). Also, MMP-2 and MMP-14 cleavage of ECM components such as laminin-5 reveal cryptic sites that act as chemotactic factors that trigger invasion as well as activate signaling via the EGF receptor (Giannelli *et al*, 1997; Schenk *et al*, 2003). The acquisition of mesenchymal characteristics by epithelial cells represents a key step in epithelial tumour progression. Matrix metalloproteinases are important for the initiation and propagation of this differentiation programme.

## CELL DEATH

Evasion of apoptosis permits cancer progression by allowing cell survival despite unfavourable conditions. Through proteolysis of various biologically active molecules, MMPs can positively or negatively regulate apoptosis in a context-dependent manner. MMP-7 releases membrane-bound Fas ligand, thereby triggering apoptosis upon binding with the Fas receptor (Powell *et al*, 1999). On the other hand, MMP-7 can inhibit apoptosis by proteolytic generation of mature HB-EGF that promotes cell survival by stimulating the ErbB4 tyrosine kinase receptor (Yu *et al*, 2002). MMP-3 induces apoptosis when overexpressed in mammary epithelial cells (Alexander *et al*, 1996), whereas MMP-11 overexpression decreases spontaneous apoptosis in tumour xenografts (Wu *et al*, 2001).

The role of MMPs in influencing apoptosis is further complemented by studies on the role of TIMPs in apoptosis. Generally, TIMP-1, TIMP-2, and TIMP-4 have been reported to exert antiapoptotic effects, while the role of TIMP-3 in apoptosis has proven complex. TIMP-1 inhibits mammary epithelial apoptosis in transgenic mice (Alexander *et al*, 1996) and in human breast cell lines (Li *et al*, 1999). TIMP-2 overexpression affords B16F10 melanoma cells protection from apoptosis (Valente *et al*, 1998). Similarly, TIMP-4 can protect human breast cancer cells from apoptosis (Jiang *et al*, 2001). Distinct from these TIMPs, TIMP-3 can either enhance or suppress apoptosis. Adenoviral infection studies have shown that high levels of TIMP-3 promote apoptosis in several cell lines. However, the loss of TIMP-3 function also accelerates apoptosis in the involuting mammary gland (Fata *et al*, 2001). It has been suggested that TIMP-3 overexpression stabilises death receptors sensitising the cell to apoptosis (Smith *et al*, 1997) or activating the mitochondrial apoptotic pathway in a FADD-dependent manner (Bond *et al*, 2002). The mechanisms by which individual TIMPs exert pro- or antiapoptotic effects will become clearer once the relevant MMP or ADAM substrates are identified. The relative levels, tissue specificity, and spatiotemporal expression of MMPs, TIMPs, and ADAMs will also influence the extracellular proteolytic axis. The sum of these factors would then determine the commitment to programmed cell death.

## CELL DIVISION

The development of cancer requires an escape from the natural breaks that keep cell division in check or oncogenic stimuli often involving growth factors and their receptors, causing aberrant cellular division. Studies using genetically modified mice have revealed that generally upregulation of MMPs promote tumour formation and growth, whereas loss of MMP expression or increased expression of their inhibitors attenuates tumorigenesis. For example, in mice with the APC^min^ mutation, genetic deletion of MMP-7 slows the formation of intestinal adenomas (Wilson *et al*, 1997). Several chemical carcinogenesis models have been used to study MMPs in the early events of tumorigenesis. MMP-1 transgenics show increased susceptibility to skin carcinogens (D'Armiento *et al*, 1995), while MMP-9 null mice show decreased tumour incidence in another skin carcinogenesis model (Coussens *et al*, 2000). In the latter case, despite the deceased tumour incidence, tumours that do form are high-grade and more aggressive. Strikingly, transgenic mice that overexpress MMP-3, MMP-7 or MMP-14 give rise to spontaneous mammary hyperplasias and malignancies (Rudolph-Owen *et al*, 1998; Sternlicht *et al*, 1999; Ha *et al*, 2001) supporting a causal role for MMPs in cancer development. Overall, trends between MMP expression and tumorigenesis are not as straightforward as initially thought.

The role of MMPs in early neoplastic lesions can also be inferred through studies of TIMPs, which should show opposing effects. TIMP-1 expression was shown to slow chemical skin carcinogenesis (Buck *et al*, 1999) and when coexpressed in MMP-3 transgenics, inhibited the formation of spontaneous preneoplastic and neoplastic lesions (Sternlicht *et al*, 1999). Moreover, TIMP-1 also effectively inhibits the formation of hepatocellular carcinoma in mice with liver expression of SV40 T antigen (CRP-TAg). Conversely, antisense expression of TIMP-1 enhances liver tumour development (Martin *et al*, 1996) as well as augments mammary epithelial proliferation (Fata *et al*, 1999). However, this phenomenon is not as simple as it first appears. Certain studies have suggested that TIMPs may function independently of their MMP-inhibitory activity. For example, TIMP-1 expression in APC^min^ mice had no effect or augmented intestinal adenomas, although a synthetic MMP inhibitor was effective in slowing tumour formation (Heppner-Goss *et al*, 1998). Thus, despite tumour attenuation by inhibition of general MMP activity, TIMP-1 did not mimic this effect and, in fact, could promote tumour growth. Other systems have also shown that TIMP gene delivery can stimulate tumorigenesis (Guedez *et al*, 2001; Jiang *et al*, 2001). This highlights the context-dependent effects of MMPs and TIMPs during cancer development.

Key to the divergent and complex roles of MMPs and TIMPs on cell proliferation is the discovery that metalloproteinases affect the bioavailability of numerous growth factors and cytokines (Table 1

**Table 1:** List of growth factor and cytokine substrates of MMPs and ADAMs

**Metalloproteinase**	**Growth factor/cytokine targeted (released from)**	**Reference**
MMP-1	IGF (IGFBP2)	Vu and Werb (2000)
	(IGFBP3)	Vu and Werb (2000)
	(IGFBP5)	Vu and Werb (2000)
	FGF (perlecan)	Vu and Werb (2000)
	TNF-*α*	Gearing *et al* (1994)
		
MMP-2	IGF (IGFBP3)	Vu and Werb (2000)
	(IGFBP5)	Vu and Werb (2000)
	IL1-*β*	Vu and Werb (2000)
	TGF-*β* (decorin)	Imai *et al* (1997)
	(LAP)	Yu and Stamenkovic (2000)
	TNF-*α*	Gearing *et al* (1994)
		
MMP-3	HB-EGF	Egeblad and Werb (2002)
	FGF (perlecan)	Vu and Werb (2000)
	IGF (IGFBP3)	Vu and Werb (2000)
	IL1-*β*	Vu and Werb (2000)
	TGF-*β* (decorin)	Imai *et al* (1997)
	TNF-*α*	Gearing *et al* (1994)
		
MMP-7	TGF-*β* (decorin)	Imai *et al* (1997)
	TNF-*α*	Gearing *et al* (1994)
		Haro *et al* (2000)
	HB-EGF	Egeblad and Werb (2002)
		
MMP-9	IGF (IGFBP3)	Vu and Werb (2000)
	IL1-*β*	Vu and Werb (2000)
	TGF-*β* (LAP)	Yu and Stamenkovic (2000)
	TNF-*α*	Gearing *et al* (1994)
	VEGF (unknown)	Bergers *et al* (2000)
	sKitL	Heissig *et al* (2002)
		
MMP-11	IGF (IGFBP-1)	Manes *et al* (1997)
MMP-12	TNF-*α*	Egeblad and Werb (2002)
MMP-14	TNF-*α*	Egeblad and Werb (2002)
MMP-17	TNF-*α*	English *et al* (2000)

). For example, MMPs cleave growth factor binding proteins that sequester and prevent growth factors from binding their receptors. In CRP-TAg mice, TIMP-1 is able to inhibit neoplastic hepatocyte proliferation by affecting MMP cleavage of insulin-like growth factor binding proteins (IGFBPs). This subsequently prevents the circulating, abnormal levels of IGF-II from binding to the type I IGF receptor and activating downstream proliferative signals (Martin *et al*, 1999). Alternatively, MMPs can release ECM-bound mitogens from the surrounding matrix such as TGF-*β* (Imai *et al*, 1997). TGF-*β* is further activated by cleavage of its binding protein, latent activating protein (LAP), by MMP-2 and MMP-9 (Yu and Stamenkovic 2000). In fact, MMP-2 deficiency phenocopies the expression of a dominant-negative TGF-*β* receptor (Vu and Werb, 2001). Growth factor signalling can also be controlled by protease-mediated receptor cleavage that sheds the ectodomains. For example, MMP-2 can release fibroblast growth factor receptor-1 (FGFR-1; Levi *et al*, 1996). Other factors, including cytokines such as tumour necrosis factor-alpha (TNF-*α*), also require cell-surface shedding. Several MMPs have been shown to cleave TNF-*α* as well as the TNF-*α* convertase (ADAM-17), which is the primary enzyme that mediates this process (Black *et al*, 1997). Since upregulation of growth factors is linked to tumorigenesis, understanding these emerging biological connections that link the MMP/ADAM/TIMP proteolytic axis to growth factor signalling is crucial in identifying appropriate and effective therapeutics for cancer treatment.

## PERSPECTIVE

From their initial discovery in tadpole tail resorption, MMPs have catapulted into the spotlight. Initially known to be important in metastatic dissemination, MMPs have emerged as regulators of fundamental cellular processes. Their historic characterisation as ECM remodelling enzymes is perhaps simply the edge of their complex biological function and recent discoveries make it necessary to reflect and reassess current strategies for MMP inhibition in cancer. Along with the discovery of ADAMs that are also inhibited by MMP inhibitors, the discovery of new MMP substrates and mechanisms of action point to the adverse interference of normal biological processes by broad-spectrum inhibitors. Matrix metalloproteinases are no longer just mediators of metastasis during late-stage malignancy, but affect tumour initiation and growth through the loss of cell adhesion, evasion of apoptosis, and deregulation of cell division. Even more perplexing are the opposing roles of TIMPs on tumour growth and apoptosis that may depend on their relative levels, the cellular context, and the developmental programme. The challenge is to design inhibitors that are highly targeted to specific proteolytic mechanisms that stifle different stages of tumour progression, while preserving the normal tissue function. Continued research into these mechanisms will prove both challenging and exciting.
